# Inside the Cell: Integrins as New Governors of Nuclear Alterations?

**DOI:** 10.3390/cancers9070082

**Published:** 2017-07-06

**Authors:** Elena Madrazo, Andrea Cordero Conde, Javier Redondo-Muñoz

**Affiliations:** 1 Section of Immuno-oncology, Instituto de Investigación Sanitaria Gregorio Marañón, 28007 Madrid, Spain; elenamdrz@gmail.com; 2 Department of Immunology, Hospital 12 de Octubre Health Research Institute (imas12), Complutense University School of Medicine, 28040 Madrid, Spain; ancorder@ucm.es

**Keywords:** integrins, nucleus, nuclear deformability, tumor microenvironment, ECM, chromatin, nuclear mechanics

## Abstract

Cancer cell migration is a complex process that requires coordinated structural changes and signals in multiple cellular compartments. The nucleus is the biggest and stiffest organelle of the cell and might alter its physical properties to allow cancer cell movement. Integrins are transmembrane receptors that mediate cell-cell and cell-extracellular matrix interactions, which regulate numerous intracellular signals and biological functions under physiological conditions. Moreover, integrins orchestrate changes in tumor cells and their microenvironment that lead to cancer growth, survival and invasiveness. Most of the research efforts have focused on targeting integrin-mediated adhesion and signaling. Recent exciting data suggest the crucial role of integrins in controlling internal cellular structures and nuclear alterations during cancer cell migration. Here we review the emerging role of integrins in nuclear biology. We highlight increasing evidence that integrins are critical for changes in multiple nuclear components, the positioning of the nucleus and its mechanical properties during cancer cell migration. Finally, we discuss how integrins are integral proteins linking the plasma membrane and the nucleus, and how they control cell migration to enable cancer invasion and infiltration. The functional connections between these cell receptors and the nucleus will serve to define new attractive therapeutic targets.

## 1. Introduction

The nucleus separates and encloses the genome of the cell from the cytoplasm and this is critical for the normal functions of eukaryotic cells. Cancer cells present an aberrant nuclear morphology compared to normal cells, including nuclear invaginations, irregular shape and volume, aberrant chromatin regions and nuclear bodies and multilobulation [1,2,3]. Moreover, the nuclear disorganization in cancer cells is used by pathologists for diagnosis and prognosis of cancer and to determine the grade of malignancy [4]. As defects in nuclear components do not directly lead to cancer development (although they are connected with other human pathologies) [5,6], we can suppose that disrupted nuclear organization in cancer cells is a consequence of cancer transformation and progression.

The nucleus is composed of a nuclear envelope (NE), which contains the outer and inner nuclear membranes and the nuclear pore complex. Underlying the NE is the nuclear lamina network, which is mainly composed of lamins and lamin-associated membrane proteins, which connect lamins to the chromatin and cytoskeleton [7]. Lamins are nuclear intermediate filaments grouped into A-type and B-type lamins that control the nuclear architecture and are linked to gene expression and global chromatin organization [8,9]. The nuclear lamina network includes several components that connect the nuclear lamina with the chromatin and the cytoskeleton. These include the LINC (Linker of nucleoskeleton and cytoskeleton complex), composed by nesprin and SUN proteins, titin, emerin and all-spetrin. Nesprins and SUN proteins are NE transmembrane proteins that connect with actin (nesprin-1 and -2), plectin (nesprin-3), and dynein and kinesin (nesprin-4 and KASH5). Other lamin-interacting proteins are associated with chromatin regions, such as lamin B receptor (LBR) and LAP2 [9]. Finally, other proteins play a role in both the cytoskeleton and the nucleoskeleton. For instance, actin and its associated proteins, such as Wiskott-Aldrich syndrome protein (WASP), focal adhesion kinase (FAK), actinin, Arp proteins, myosin, ERM (Ezrin, Radixin, Moesin) with others, shuttle between the nucleus and the cytoplasm. The nuclear fraction of these proteins links to the epigenetic machinery, the lamina network and regulate some nuclear functions [10,11,12,13,14].

Chromatin is composed of the deoxyribonucleic acid (DNA) and its associated proteins and molecules. Chromatin structure is highly coordinated to regulate gene expression, cell-cycle, DNA damage, cell development and differentiation [15]. Chromatin is condensed and relaxed at different nuclear regions according to epigenetic alterations. These epigenetic changes comprise post-translational modifications of the DNA (methylation), histone proteins (methylation, acetylation, ubiquitylation, SUMOylation) and non-coding RNA sequences, which regulate chromatin structure, function and contribute to the nuclear architecture [15]. Cancer cells manifest many epigenetic abnormalities, which lead to genomic instability and aberrant gene expression during cancer progression and recurrence. Due to the aberrant nuclear architecture of cancer cells, nuclear changes have been proposed a hallmark of cancer and may lead to the identification of new therapeutic targets [16,17].

## 2. Integrins and Cell Migration

Integrins are heterodimeric receptors consisting of two subunits (α and β) described more than 30 years ago [18,19]. This receptor family comprises 24 integrin heterodimers in vertebrates, formed by the combination of 18 α and 8 β subunits that “integrate” chemical and physical stimuli from the extracellular matrix (ECM), other cells and microenvironment into the cytoplasm and nucleus of the cell [20,21]. Integrins are type 1 transmembrane receptors with a common structure: an extracellular domain, a transmembrane region and a cytoplasmic tail, which associates with cytoskeletal components such as talin, paxillin, tensin and actinin; and signaling transducers such as FAK, integrin linked kinase (ILK), cytohesin-1 and the cytoplasmic domain associated protein-1 (ICAP-1) [22,23]. Integrins play a critical role in several cellular functions, including adhesion, migration, immune synapse and signaling, proliferation, survival, thrombocytosis, angiogenesis and cell differentiation [24,25]. Due to these diverse functions, integrins are implicated in many human pathologies including inflammation, autoimmune and infection diseases, and are fundamental also in cancer [26,27].

There is a clear link between oncogenes and integrin expression. For instance, Boudjadi et al. described that α1β1 expression is regulated by the oncogene myc in colorectal cancer cells [28] and β1 integrins-mediated adhesion appears to drive cancer cell proliferation and metastasis in some contexts [29]. In breast cancer cells, integrin signaling via PI3K (Phosphoinositide 3-kinase) and FAK controls the translocation of Bax to the mitochondrial membrane, thereby repressing apoptosis [30]. A similar cytoprotective effect is observed in hematological cancers when cells are retained in microenvironmental niches [31,32]. Several groups have described how integrins and their focal adhesion (FA) partners also regulate apoptosis by modulating the oncogene *p*53. For instance, integrins regulate *p*53 expression and response while FAK interacts with *p*53 and regulates its expression in several cancer type cells, including melanoma and sarcoma cells [33,34].

Integrins are used by cancer cells to control growth factor signaling [35]. It is proposed that the recycling machinery controls the presence of α5β1 and EGFR1 to promote cell migration of ovarian carcinoma cells [36] and Zahir et al. showed that α6β4 controls NF-kB activity via EGFR1/Rac in breast tumors [37]. TGFβ (tumor growth factor β) is a promoter of epithelial mesenchymal transition (EMT) and in turn, EMT and its signaling is regulated by the matrix stiffness and the action of metalloproteinases associated with integrins. For instance, MT1-MMP and αvβ8 regulate TGFβ signaling in several cancer cell types, including colon adenocarcinoma, fibrosarcoma, lung carcinoma, hepatocarcinoma, etc. [38,39,40]. Intriguingly, cancer stem cells (CSC) present specific integrin expression patterns, including α6, β1, and β3, which appear to help to maintain the CSC population [41]. CSC are a subpopulation of cancer cells that possess self-renewal properties and tumor initiating capacity. It has been recently reviewed that integrins control their stem cell characteristics in the stem cell niche [41].

Given their diverse roles in human disease, integrins have increasingly been viewed as therapeutic targets. Approaches include targeting the integrins or their ligands by using antibodies, peptides and organic inhibitors (see Table 1 for more information). For instance, blocking strategies have been employed against αIIbβ3 (thrombosis), α4β1 (multiple sclerosis), α4β7 (Crohn disease and colitis), and others [42,43,44,45,46,47,48,49]. In cancer, some integrin blocking therapies are under clinical development, such as the anti-integrin antibodies, antagonists and small molecules against αvβ3, αvβ5, α5β1 and αvβ6 [50,51,52,53,54,55,56,57,58,59,60]. However, despite the promising preclinical data, clinical trials failed to show any improvement over conventional therapies and so research efforts are currently focused on developing new tumor targeted drug-delivery strategies approach to overcome clinical limitations.

### Integrins and Cell Migration

A critical function of integrins is their control of cell adhesion and migration. Integrins regulate membrane trafficking and endocytosis, epithelial-mesenchymal transition (EMT), cell polarization, cytoskeletal rearrangements, activity and localization of matrix metalloproteases, and the interplay between cancer cells and their surrounding cancer microenvironment [26].

Cancer cell migration is a complex process involving many molecular interactions. However, integrins orchestrate at least some of these processes including the interaction between cell receptors and the ECM, and the integration of signals that modulate migration rate and cell phenotype. It is worthy of note that migration in the 3D ECM presents substantial differences to 2D in vitro culture conditions. Most notably, nuclear deformability appears to be the limiting factor for effective cell migration in these more confined physiological conditions [61]. In general, integrins are fundamental for normal and cancer cells to sense the mechanobiological signals from the microenvironment [62]. Several modes of cancer cell migration in confined conditions have been reported, such as collective and single cell migration and movement through specific ECM regions acting as migrating tracks [62]. In the case of single cell migration, receptor adhesion and actomyosin contractility are critical determinants of these migration types, which include lobopodial, lamellipodial, “fibroblast-ameboidal” (A1), Bleb-ameboidal (A2), and osmotic engine models [62,63]. Moreover, depletion of integrin, talin or vinculin did not affect specific cell morphologies [64], suggesting that blocking integrins might induce switch between these phenotypes and reduces the cell migration. These molecular mechanisms have been recently reviewed and are not subject of this review.

One remarkable aspect of the 3D environment surrounding cancer cells is that it is often altered, both in terms of the specific cellular constituents present and the organization of the cellular microenvironment, when compared with normal ECM. For instance, in breast cancer there is a correlation between the ECM stiffness and the malignant and invasive phenotype of mammary epithelial cells [65,66]. The integrin “adhesome” has been extensively studied and divided according to the FAK/Paxillin, Talin/Vinculin, ILK/Kindlin, and α-Actinin/Zyxin pathways [67]. These specific integrin adhesome molecular partners are promising therapeutic targets to reduce cancer invasion [68]. However, how these molecules control nuclear changes in migrating cancer cells is yet to be fully elucidated.

Despite the canonical function of integrins during cell migration in 2D, cancer cells can use integrin-dependent and independent mechanisms to migrate under 3D conditions. For instance, blocking or knocking-out integrins and their associated partner molecules does not affect cell migration of normal immune cells and leukemic cells in 3D conditions in vitro and in vivo [69,70,71]. In spite of this, other evidence illustrates that integrins might be involved in immune cell infiltration, demonstrating that both integrin-dependent and independent migration is possible under different conditions [72,73,74]. For cancer cells, integrins and their associated glycoproteins have been shown to support cancer cell adhesion and contribute to tumor progression and invasive cancer phenotypes [75]. It is broadly known that integrins are critical for cancer cell migration in confined spaces; although it has been proposed that carcinosarcoma cells are capable of integrin-independent migration [76]. Integrins and their FA partners drive ECM remodeling and intracellular signals such as PI3K and FAK [77,78]. Remarkably, integrin recycling and endocytosis also drives cancer cell invasion in 3D environments. For instance, Casswell et al. showed that α5β1 recycling via Rab25 drives cancer cell migration through 3D collagen gels [79]. Both the stroma and cancer cells control the ECM rigidity, which promotes tumor invasion, invadopodia formation and metastasis via EMT of breast cancer cells induced by the nuclear factor TWIST1 [80] and EMT of epithelial cells by modulating TGF signals [40]. The significance of ECM stiffness and its mechanical properties as has been reviewed recently [81]. This review suggested that these physical properties of the ECM represented promising therapeutic targets. Herein, it is mandatory to explore new functional connections driven by integrins and their associated molecules during cancer cell migration.

## 3. The Nucleus and Cell Migration

In order to metastasize, cancer cells have to move through endothelial barriers, stiff ECM and different confined microenvironments [82]. While migration in 2D does not present space restrictions, in 3D conditions cells must alter their nucleus to facilitate their migration through confined spaces such as interstitial spaces or transendothelial migration [83,84,85]. Over the last few years, the notion of biomechanics and its significance on cell migration and tumor dissemination has gaining research interest. As the nucleus is physically connected to the cytoskeleton and the plasma membrane, the active interplay between the nucleus and the cell body is fundamental during cell migration (Table 2). Therefore, the regulation of the nuclear shape, position and deformation is critical in controlling the migration of both normal and cancer cells.

### 3.1. Nuclear Translocation and Rotation

The nucleus has to move coordinately with the cell body, according to cellular tensegrity [86]. This concept highlights the intracellular filament network that connects cell poles and organelles [86]. The nucleus might need to rotate in order to align with the axis of migration and behind the centrosome of a polarizing cell. This nuclear rotation depends on microtubules and actomyosin contractility and helps the cell to polarize and move through confined spaces [87]. Therefore, cytoskeletal rearrangements drive the cell polarity, the leading and trailing edges and internal dynamics. On the other hand, LINC acts as a nuclear node that transmits forces between the cytoskeleton and the nucleus, and its critical for nuclear positioning, reorientation and centrosome attachment to the nucleus [88]. Swift et al. described how the nuclear translocation is crucial during mesenchymal stem cell migration [89]. It has been described that nuclear localization has to coordinate with the cell body via actomyosin, which applies pulling and pushing forces on the nucleus rather like a piston. The piston mechanism is a key factor for lobopodial movement of fibroblasts and fibrosarcoma cells [90]. Thomas et al. have described how the myosin IIA cooperates with vimentin to regulate myosin contractility at the leading edge of breast cancer cells to pull the nucleus [91]. Myosin IIB localizes at the perinuclear actin rim and the trailing edge, driving nuclear pushing from the cell rear through ECM constrictions [91]. Microtubules and their associated proteins dynein and kinesin also contribute to nuclear rotation and pulling during cell migration, as reported in myoblasts and neurons [92,93,94]. LINC complexes are critical to control and keep the balance of the PMT (point of maximum tension) for the nucleus of migrating fibroblasts [95]. Furthermore, it has been shown that integrins localize at the leading edge of migrating cells and control actomyosin contractility through the Rho/ROCK axis [96]. The functional connections between cytoskeleton polarization, internal forces transmitted by LINC complexes and the nuclear forces applied need to be explored to define precisely how cohesive forces control nuclear positioning in cancer cells.

### 3.2. Nuclear Deformability

As we introduced previously, nuclear deformability is critical to allow cancer cell migration in confined conditions [36]. During cell migration, the nucleus becomes more malleable, and sensitive to forces applied from the cytoskeleton. Multiple nuclear components determine the nuclear deformability, including lamins, the chromatin and nucleoplasm. The nuclear lamina is the major contributor to the stiffness and the mechanical properties of the nucleus of lung carcinoma, glioblastoma and mesenchymal stem cells [89,97]. Lamin A expression and its stoichiometry with respect to lamin B appear to control nuclear stiffness and mechanics [97]. For instance, expression of lamin A is required for proper nuclear movement, downregulation of E-Cadherin, increase CSC phenotype and cell migration efficiency in colorectal cancer [98]. Kong et al. described that lamin A overexpression correlates with PI3K/AKT signaling pathway and the malignant behavior of pancreatic cells [99]. There is evidence that the migration of cancer cells (such as melanoma and neuroblastoma cells) requires reduced levels of lamin A, as lower nuclear stiffness correlates with higher deformability of the nucleus [100,101]. Furthermore, leading cells at the periphery of tumor xenografts present low lamin A levels suggesting more invasive phenotype than cells located at the center of the tumor [97]. These controversial findings might be explained by different migration types as well as other nuclear contributors to nuclear deformability and migration. This idea agrees with other publications that present how defective lamin A expression is linked to chromatin structure, LINC expression and localization and cohesive nuclear-cytoskeleton connections in fibroblast and osteosarcoma cells [102,103]. Loss of other nucleoskeletal components, such as emerin, also affects the nuclear shape and mechanics of thyroid carcinomas [104].

It is now well established that cell migration is associated with epigenetic changes. DNA methylation and histone modifications related to heterochromatin (H4K20me1, H3K27me3 and H3K9me3) are upregulated in several invasive cancer cell types, including breast, ovarian and melanoma cancer cells [105,106,107]. Likewise, changes in histone 1 mobility are also related to cell migration [105]. Chromatin compaction might be coupled to the cytoskeleton to facilitate efficient nuclear positioning and reshaping and to foster biomechanical connections [108,109]. Epigenetic changes contribute directly to the mechanical properties of the nucleus and are present in specific genomic regions linked to lamins and other nuclear envelope components (lamina-associated domains, LADs) [110]. This aligns with the novel non-genomic functions assigned to epigenetic changes by Bustin and Misteli [111]. Furthermore, treatment with the methyltransferase inhibitor MTA (5′-deoxy-5′-methylthioadenosine) reduced chromatin condensation, proliferation and invasion of bladder cancer cells [112]. In gastric carcinoma cells, the use of decitabine, a DNA methyltransferase inhibitor, impaired cell migration, suggesting this approach as a potential therapeutic strategy against cancer dissemination [113]. Recently, Maizels et al. demonstrated that tumor progression leads to chromatin plasticity, which in turn controls the invasiveness and proliferation of melanoma cells [114]. Although high heterochromatin levels increase the nuclear stiffness and might impair cancer cell migration, they also facilitate nuclear reshaping and effective cytoskeletal forces during nuclear translocation. Recently, it has been described that normal and cancer cells present NE rupture and DNA damage during cell migration across narrow 3D spaces [115,116]. This requires the induction of the DNA damage repair machinery and the endosomal sorting complexes required for transport (ESCRT) III complex and might contribute to genomic instability in cancer cells. This highlights how chromatin structure might compromise cancer cell migration. Moreover, Irianto et al. have described how osteosarcoma cells migrating through narrow spaces present chromatin compaction and defective mobility of DNA repair factors and nucleases [117].

Finally, instead of internal nuclear components, such as lamins, LINC complexes and the chromatin, it has been suggested that the cytoplasmic perinuclear cytoskeleton may regulate nuclear deformability through confined conditions. CiAN (confinement induced actin network) define several components such as Arp2/3 and fascin that surround the nucleus and manage its deformation in immune and cancer cells [118,119]. This opens new research questions and potential therapeutic targets to explore in a future.

### 3.3. Intranuclear Structures

Cell-ECM interactions can lead to the redistribution of intra-nuclear structures. These nuclear regions might influence nuclear deformability and cell capacity to invade and migrate under constricted conditions. The nucleolus is an ultrastructure composed by multiple proteins and RNAs that regulate nuclear functions such as gene transcription [120]. It has been reported that the nucleolus localizes opposite the MTOC (Microtubule organizing center) in migrating *Dictyostelium discoideum*, and this localization depends on microtubules [121]. During development, columnar cells alter the size and structure of the nucleolus according to their migration to villus top [122]. Other internal nuclear structures reorganized by cell-ECM interactions include Cajal bodies, which are highly mobile and can interact with the nucleolus in HeLa cells, as presented by Platani et al. [123].

In addition to nuclear lamina and the chromatin structure, other cytoskeletal components localize in the nucleus and contribute to the nuclear deformability in regulating cell invasiveness. For instance, Rac1 is imported to the nucleus, where Rac controls nuclear shape and chromatin organization and promotes an invasive phenotype of prostatic cancer cells [124]. Other nuclear and cytoskeleton components regulate chromatin structure; for instance, WASP and FAK translocate from the cytoplasm into the nucleus where they control and interact with specific epigenetic remodelers and histone modifications, such as the protein MBD2 (methyl CpG-binding protein 2) [10,12]. Also nuclear ERM proteins control chromatin conformation. For instance, specific phosphorylations of ezrin drive its nuclear localization, which might influence on gene transcription, nuclear shape and mechanics of osteosarcoma cells [13]. Finally, despite cell tensegrity new evidence demonstrates how the nucleus presents its own mechanical properties. Guilluy et al., showed that nesprin-1 controls the mechanical response of isolated nuclei to external forces, by regulating emerin phosphorylation [108]. This supports the idea that the nucleus is a mechanosensitive organelle independent of the cytoplasm.

### 3.4. Role of Integrins in Nuclear Modifications

Integrins are critical for cancer cell migration. Therefore, deciphering their role in nuclear changes and mechanics should expand our knowledge about the molecular mechanisms used during tumor cell migration and metastasis. Migrating cells relocalize several cell components, including MTOC, mitochondria, Golgi apparatus. Currently, we know that integrins drive cell organelle positioning. For instance, the α5β1 integrin, via the protooncogene tyrosine-protein kinase Src and MLCK (myosin light chain kinase) activity, controls Golgi distribution in fibroblasts [125]. It has been reported that in migrating T lymphocytes the integrin LFA-1 (αLβ2) is critical for the redistribution of CG-NAP/AKAP450 protein (centrosome and Golgi localized protein kinase N-associated AKAP protein) [126]. Regarding centrosome positioning, Hurtado et al. showed how delocalization of the Golgi impairs the polarity axis and migration directionality in epithelial cells [127]. Also, the centrosomal component, AKAP350 contributes to non-immune cell migration, by controlling the centrosome positioning in hepatocellular carcinoma, liver adenocarcinoma and epithelial cells [128]. Other organelle redistribution is also controlled by integrins such as mitochondrial relocalization is driven by Miro-1 and dynein during lymphocyte movement and transendothelial migration [129]. Moreover, Miro-1 and other mitochondrial regulators, such as SNPH (syntaphilin) and kinesin KIF5B, have been defined as cell invasion regulators that connect mitochondria with the cytoskeleton in multiple normal and cancer cell types, including fibroblasts, glioblastoma, prostate cancer and breast adenocarcinomas [130].

**Figure 1:**
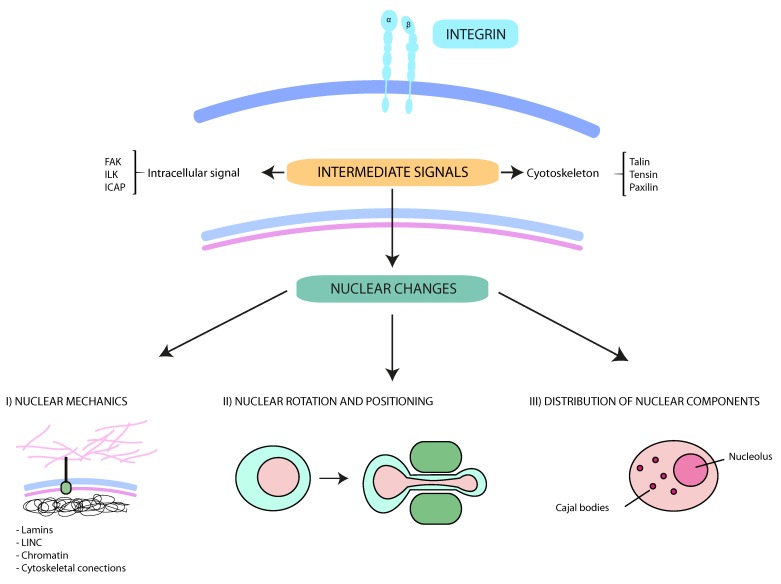
Schematic representation of the interplay between integrins and nuclear changes. Multiple external stimuli from the tumor microenvironment control integrin-mediated signals. The cytoskeleton and protein kinases transmit these signals from the cell surface into the nucleus. These downstream pathways contribute to (I) nuclear mechanics and deformability (mainly regulated by lamins, LINC (Linker of nucleoskeleton and cytoskeleton) complexes, the perinuclear cytoskeleton and the chromatin); (II) the rotation and movement of the nucleus (critical for specific cell migration types, such as lobopodial migration); and (III) the intranuclear disposition. Together, the interplay between integrins and nucleus support cancer cell migration and dissemination through constricted conditions.

More than 20 years ago, a connection between integrins, cytoskeleton and nucleus was described [131]. Since then, novel reports have described how integrin dependent adhesion and signaling govern the positioning and properties of intranuclear components and superstructures during cell migration (see Figure 1). For instance, Poh et al. described that mechanical forces applied through integrins redistribute the interaction and localization of the Cajal Bodies components coilin and SMN in fibroblasts and HeLa cells [132]. Additionally, actin cytoskeleton and the nuclear lamina, but not microtubules, are critical for force transmission from the integrins into Cajal bodies, supporting an integral signaling network between integrins and nuclear structures [132]. More recently, it has been unraveled that 3D ECM controls the number of nucleoli and nuclear changes in breast cancer cells, and these changes are β1 integrin dependent [133].

Integrins are crucial promoters of epigenetic changes that might contribute to nuclear deformability. For instance, there is a clear interplay between integrins and their associated proteins with histone methyltransferases. The depletion of these epigenetic components impairs proper cell adhesion and cell cycle progression. For instance, EZH2, a key component of H3K27 methylation, regulates cofilin and talin activity and drives the cell migration of lymphocytes and colon carcinoma cells [134,135]. Also knockdown of mDPY-30, RbBP5 and other subunits of the H3K4 methyltransferase complex regulates the endosomal recycling system and induces cell protrusions and promotes invasion [136]. This might be connected to integrin trafficking and the ability of cancer cells to recycle cell receptors, and must be addressed in a future.

On the other hand, cell adhesion via integrins promotes epigenetic changes that influence on the nuclear behavior. It has been reported that α4β1 integrin affects H3K9 methylation and nuclear stiffness induced by G9a in lymphocytes. Moreover, inhibition or depletion of G9a abrogates cell migration [137]. Accordingly, myeloma cell adhesion (presumably mediated by α5β1 and α4β1 integrins) upregulates several epigenetic modifications that protect cells from apoptosis [138]. Aligning with this, it has been reported that fibroblasts regulate actomyosin and cytoskeletal forces in respond to ECM and constricted conditions, which affect lamin A expression, heterochromatin levels and telomere dynamics [84]. Other FA components act as fundamental mechanotransductors to transmit external forces into nuclear changes, including gene expression. For instance, cell culture in stiff ECM leads to the translocation of YAP (Yes associated protein) into the nucleus to active SMAD factors transcription of breast and squamous cell carcinoma [139]. Also the interplay between integrins and their FA partners and the regulation of DNA repair molecules must still be explored to determine new mechano-therapies [115]. Together, this suggests that integrin-mediated adhesion control chromatin dynamics and epigenetic changes that regulate gene expression, nuclear mechanics and cell migration.

As we discussed in the previous section, the nucleus must rotate and move according to the cell body and the dynamic contractile forces applied by the actomyosin cytoskeleton. Remarkably, inhibition of αvβ3 and β1 integrins, blocks the internal pressure in fibroblast lobopodial migration [140]. This nuclear mechanism requires nesprin-3 and lamin A during this integrin-dependent adhesion [140]. The levels of β3 integrins depend on the external stimuli and metastatic microenvironment of several cancer cell types, including adenocarcinoma, breast cancer and squamous lung cancer [141,142]. This suggests that cancer cells might regulate the expression of β3 integrins to switch to nuclear piston cancer cell invasion or the canonical ECM degradation via MMPs.

Together these results explore the molecular and functional connections between the integrins and the nucleus, and support the idea that integrins might act as cellular and nuclear governors during cancer cell migration.

## 4. Conclusions and Perspectives

Over the past years, research has focused on the role of integrins during cell migration. Research has shown that integrins govern, through multiple intracellular pathways, many physiological processes including cell adhesion, survival, cell cycle and migration. This has led to a deeper understanding of their contribution to cancer progression and the development of several therapeutic strategies for the treatment of cancers and other human diseases. Unfortunately, clinical trials targeting cancer metastasis using integrin inhibition did not yield successful results. The challenge now is to define and identify novel therapeutic targets linked to integrins that might serve as more effective therapeutic opportunities. Once such potential opportunity comes from the insight that integrins play a critical role in modulating the physical structure of the nucleus in order to promote the movement of cancer cells through interstitial spaces and constricted environments. It is well known that cancer cells migrate and colonize of metastatic niches, which act as sanctuaries to protect cancer cells from existing chemo- and radiotherapies. In this review, we discussed the expansion of our knowledge of integrin-mediated confined migration with a view towards using this knowledge to develop novel treatments and improve the effectiveness of conventional therapies.

New advances in our understanding have shed light on how the physical shape and structure of the nucleus controls the migration of normal and cancer cells. Nuclear components, such as lamins, histones and nucleoskeletal proteins can change the ability of cells to translocate their nuclei or modify their mechanical properties to squeeze through narrow spaces. Here, we have reviewed some recent discoveries describing functional links between integrins with nuclear components and changes in nuclear deformability. Integrins are directly involved in the three main nuclear changes explored in this review, which includes nuclear translocation, nuclear deformability and functional connections with other intranuclear structures such as the nucleolus. Beyond our current knowledge about nuclear changes induced by integrins, additional research is clearly required to identify new targets against cancer migration and infiltration. Significant open questions remain to be elucidated, regarding to the molecular mechanisms connecting integrins with nuclear changes, and whether these require only require nucleus-cytoskeletal connections or whether signaling pathways might be also involved. Still, how alterations in the nuclear lamina, chromatin conformation, nucleoli number, etc. orchestrate nuclear structure and the mechanical properties of cancer cells need to be clarified. Exploring these questions would be extremely valuable to identify specific mechanisms used by migrating cells and whether any of these are specific to cancer cells. This in turn may open up an important new field of cancer research in the future and provide novel translational treatments against cancer dissemination.

## Figures and Tables

**Table 1:** Preclinical and clinical trials evaluating drugs and inhibitors against integrins and their ligands in human disease and cancer.

Type	Class	Name	Target	Pathology	References
Integrin Targering	Monoclonal Antibody	Natalizumab	α4β1, α4β7	Multiple sclerosis	[42]
		Efalizumab	CD11a	Psoriasis	[43]
		Efalizumab	CD11a	Inflammatory bone loss (Rheumatoid arthritis)	[44]
		Natalizumab	α4β7, αEβ7	Crohn’s disease and ulcerative colitis	[45,47,48]
		Vedolizumab			
		AMD181			
		Etrolizumab			
		Abciximab	αIIbβ3	Acute coronary síndrome (ACS) undergoing Percutaneous coronary intervention (PCI)	[46]
		Vitaxin	αV, αVβ3	Cancer	[49,50,51,52]
		MEDI 522			
		Intetumumab			
		Etaracizumab			
		Volociximab	α5β1		[53,54]
	Peptide	Eptifibatide	αIIbβ3	NSTEMI with PCI	[46]
		DisBA-01	αVβ3	Cancer	[53,54]
		PHSCN	α5β1		
	Organic (Non-peptide)	AJM300	α4β1, α4β7	Crohn’s disease and ulcerative colitis	[47,48]
		Tirofiban	αIIbβ3	NSTEMI with PCI	[46]
		PSK1404	αVβ3	Cancer	[57,58,59]
		IH1062			
		GIPG0187			
		MK0429			
		GIPG0187	α5β1		[60]
		SJ749 Resveratol			
Ligand Targering	Monoclonal antibody	PF-00547659	MAdCAM-1	Crohn’s disease and ulcerative colitis	[45]
		Eldelumab	CXCL10		[47]
	Peptide	ALOS4		Cancer	[54]

**Table 2:** Overview of the nuclear changes that facilitate cell migration.

Nuclear Function	Element	Reference
Nuclear deformability	Lamin A and C	[89,97]
	Lamin B	[89,97]
	H3K9me3	[84,105,106,107,114,137,139]
	H3K27me3	
	H4K20me1	
	Perinuclear cytoskeleton	[118,119]
Nuclear disposition	Nucleolus	[121,122]
	Cajal bodies	[123]
Nuclear rotation and positioning	Microtubules	[87,93,94]
	Dynein	[93,94]
	Kinesin	[93]
	LINC complex	[86,88]
	Actomyosin	[87,89]
